# Social Media as an Early Proxy for Social Distancing Indicated by the COVID-19 Reproduction Number: Observational Study

**DOI:** 10.2196/21340

**Published:** 2020-10-20

**Authors:** Joseph Younis, Harvy Freitag, Jeremy S Ruthberg, Jonathan P Romanes, Craig Nielsen, Neil Mehta

**Affiliations:** 1 Case Western Reserve School of Medicine Cleveland, OH United States; 2 Kansas City University of Medicine and Biosciences Kansas, MO United States; 3 Department of Internal Medicine and Geriatrics Cleveland Clinic Foundation Cleveland, OH United States

**Keywords:** COVID-19, social media, Google Trends, Twitter, Instagram, reproduction number, estimated reproduction number, social distancing, public health surveillance, social media surveillance, Google Maps, Apple Maps, pandemic, epidemic

## Abstract

**Background:**

The magnitude and time course of the COVID-19 epidemic in the United States depends on early interventions to reduce the basic reproductive number to below 1. It is imperative, then, to develop methods to actively assess where quarantine measures such as social distancing may be deficient and suppress those potential resurgence nodes as early as possible.

**Objective:**

We ask if social media is an early indicator of public social distancing measures in the United States by investigating its correlation with the time-varying reproduction number (R_t_) as compared to social mobility estimates reported from Google and Apple Maps.

**Methods:**

In this observational study, the estimated R_t_ was obtained for the period between March 5 and April 5, 2020, using the EpiEstim package. Social media activity was assessed using queries of “social distancing” or “#socialdistancing” on Google Trends, Instagram, and Twitter, with social mobility assessed using Apple and Google Maps data. Cross-correlations were performed between R_t_ and social media activity or mobility for the United States. We used Pearson correlations and the coefficient of determination (ρ) with significance set to *P*<.05.

**Results:**

Negative correlations were found between Google search interest for “social distancing” and R_t_ in the United States (*P*<.001), and between search interest and state-specific R_t_ for 9 states with the highest COVID-19 cases (*P*<.001); most states experienced a delay varying between 3-8 days before reaching significance. A negative correlation was seen at a 4-day delay from the start of the Instagram hashtag “#socialdistancing” and at 6 days for Twitter (*P*<.001). Significant correlations between R_t_ and social media manifest earlier in time compared to social mobility measures from Google and Apple Maps, with peaks at –6 and –4 days. Meanwhile, changes in social mobility correlated best with R_t_ at –2 days and +1 day for workplace and grocery/pharmacy, respectively.

**Conclusions:**

Our study demonstrates the potential use of Google Trends, Instagram, and Twitter as epidemiological tools in the assessment of social distancing measures in the United States during the early course of the COVID-19 pandemic. Their correlation and earlier rise and peak in correlative strength with R_t_ when compared to social mobility may provide proactive insight into whether social distancing efforts are sufficiently enacted. Whether this proves valuable in the creation of more accurate assessments of the early epidemic course is uncertain due to limitations. These limitations include the use of a biased sample that is internet literate with internet access, which may covary with socioeconomic status, education, geography, and age, and the use of subtotal social media mentions of social distancing. Future studies should focus on investigating how social media reactions change during the course of the epidemic, as well as the conversion of social media behavior to actual physical behavior.

## Introduction

Public health measures are the epicenter of global efforts to combat the COVID-19 pandemic [1]. The premise of these measures converges on a central notion: decreasing the basic reproductive number (R_0_) of the novel coronavirus below 1 to suppress transmission. With an R_0_ value below 1, the virus can no longer sustainably propagate from one person to another, eventually halting its spread [2]. The most championed of these efforts is the idea of “social distancing,” or the practice of distancing yourself from others to reduce respiratory droplet transmission, the primary mode of transmission for COVID-19 [3]. However, social distancing has not been inconsequential, with primary concern to socioeconomic health. Several macroeconomic reports exploring the supply and demand shock of COVID-19 describe that its effects may rival that of the 1918 Spanish Flu and the Great Depression [4].

Transmission of COVID-19 was first detected in the United States on February 2020, and by mid-March, all 50 states and four US territories had reported cases of COVID-19 [5]. The total number of confirmed cases continued to rise exponentially before this trend was broken in early April. In an effort to slow transmission, several states implemented strict lockdowns, curfews, and business restrictions [6]. New York Governor Cuomo declared a state of emergency on March 7, and New York City implemented one of the first large-scale lockdowns of schools, temples, and other large gathering places in Rochelle. This further extended to include stay-at-home orders in other areas of New York, California, and Illinois. Restrictions on businesses deemed nonessential were eventually implemented in more than 40 states [6].

In response, decreases across several economic sectors have been witnessed, leading to financial strain on American households. Over 10 million unemployment claims were filed in the 2 weeks ending on March 28, 2020 [7]; for reference, the previous peak was at 695,000 claims in October 1982. National-level interventions such as mandated paid time off and a historic US $2 trillion stimulus package (Coronavirus Aid, Relief, and Economic Security Act) were used to mitigate the broad impact of COVID-19 [8].

Early intervention is ideal for the mitigation of a pandemic’s socioeconomic and health costs, but such potential is often a post hoc discovery. A more practical approach is active scrutiny and revision of the implemented measures, ideally in the early phases of the pandemic’s course [9]. Recent efforts have attempted to quantify social distancing efforts using Google or Apple Maps’ user activity [10,11]. Although these tools accurately reflect social behavior at a point in time, we hypothesize that Google Trends and social media yield earlier actionable insight that can help control the pandemic’s trajectory.

Google Trends and social media (eg, Instagram and Twitter) are used extensively in the scientific literature and have been validated against external reference data sets in numerous public health and health surveillance studies [12-17]. With an estimated 35% and 27% of all US citizens using Instagram and Twitter, respectively, on a regular basis and 89.7% of digital users searching on Google, these avenues remain the most practical tools for study [18-20]. Likewise, studies during this pandemic are investigating the utility of social media in the dissemination of preventive health information [21-23], and Twitter recently provided full access for prospective social media data tracking for COVID-19 research. Despite this, their use as epidemiological tools in the assessment of social behavior in early epidemic courses remains to be determined.

In this study, we investigate the use of Google Trends, Instagram, and Twitter as tools for the evaluation of social distancing measures by the public in the early epidemic phase. We first highlight a correlation between social distancing measures as captured by social media and national and state-specific time-varying reproduction number (R_t_), an epidemiological estimate of R_0_ throughout an epidemic. We then compare the correlation of these social media avenues with R_t_ to the correlation of Google and Apple Maps’ user activity with R_t_. We focused on the top nine affected states from the time of writing, April 10, 2020. We collected the most recent social media data using Google Trends, Twitter, and Instagram, and used the updated confirmed cases compiled by the Centers of Disease Control COVID-19 Case Data and John Hopkins Coronavirus Resource Center [24,25].

## Methods

### Database Inclusion

We used Google Trends, Instagram, and Twitter. In addition to their established use in the scientific literature, we also focused on Instagram and Twitter because their demographic overlaps significantly with the public-facing jobs [18-20] most likely to be affected by social distancing. Furthermore, a poll conducted by the Morning Consult between March 27 and 30, 2020, reported 88% of Americans between the ages of 30-54 years are practicing social distancing to some extent [20,26], an age range closely resembling Instagram and Twitter’s median ages of 34 and 40, respectively.

The choice to include only the top nine states by COVID-19 incidence was made because lower incidence states yielded insufficient social media and incidence data. When the analysis was run on the bottom nine states by COVID-19 incidence, the results displayed erratic patterns of social distancing search interest with no clear peak and days with no data, suggesting low search volumes; additionally, the R_t_ displayed large error margins and could not be calculated continuously over the study period.

### Google Trends 

Google Trends records billions of data points from search terms entered by the public. It then compares the summative search volume of each search query (defined as the exact term entered into Google’s search bar) to the day of highest search volume to yield a search volume index (SVI) score of 1-100. SVI is assigned to each day and represents that day’s relative search frequency. Google Trends contains a geo-filtering feature that allows search data from within the United States or, to be more granular, from specific states.

Google Trends data for the search query “Social Distancing” was collected on April 10, 2020, for March 1, 2020, through April 10, 2020.

### Instagram and Twitter

Instagram and Twitter are social networking platforms that can be accessed on a phone app or internet website. As of 2018, there are 107 million Instagram users in the United States. Similarly, as of January 2020, 59 million Twitter users are American, comprising the largest percentage of Twitter’s user base. Together, these social networking services capture a large percentage of the American population [20,26].

Unamo search algorithms were used to capture the historical frequency of mentions for the hashtag “#socialdistancing” in the United States on Twitter and Instagram between March 1 and April 10, 2020 [27].

### Calculation of R_t_

R_0_ is the number of individuals infected by a single infected individual during his or her entire infectious periods in a population that is entirely susceptible.

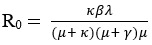



Where *κ* is the rate at which an exposed individual becomes infectious, *β* is the probability that a susceptible individual becomes infected upon interaction with an infected individual, *λ* is the birth rate of susceptible individuals, *μ* is the per capita natural death rate, and *γ* is the per capita recovery rate.

The R_0_ for COVID-19 has varied in value from 1.4 to as high as 11.1 reported from some communities in China and Singapore [28,29].

The R_t_ is an epidemiological estimate of R_0_ calculated using two variables: (1) the daily incidence of acute respiratory illness onset and (2) the distribution of the serial interval (time interval between symptoms onset in a case and in their infector).

The daily incidence of COVID-19 in the United States was obtained from estimations of symptom onset provided by the Centers for Disease Control and Prevention COVID-19 Case Data, which contains data up to April 5, 2020 [24]. The statewide incidence rate is based on confirmed cases obtained from the John Hopkins Coronavirus Resource Center. The serial interval was obtained using available parametric data computed previously for the initial outbreak of COVID-19 [30].

We used the R statistical software (R Foundation for Statistical Computing) along with the EpiEstim package to calculate the R_t_ using the aforementioned parameters for the period of March 5 to April 5, 2020. R_t_ for the United States and top nine states by confirmed COVID-19 cases was derived for this time period. For subsequent calculations, we included data after the onset of at least 100 confirmed cases in each state, as the R_t_ prior to that had standard deviations in excess of 0.5. 

The Google Trends SVI for “social distancing” was then independently compared to R_t_ for the top nine affected states (New York, California, Pennsylvania, Massachusetts, New Jersey, Florida, Louisiana, Michigan, and Illinois) and the United States as a whole. Analyses were performed using Pearson correlations with significance set to α<.05 then plotted on logarithmic graphs. Correlations were obtained using raw data and after varying periods of time delay between the Google SVI or social media mentions and changes in R_t_.

Cross-correlations for the relationship between R_t_ and measures of social distancing in the United States were performed, using available data based on Google Maps tracking that measures changes in percent social mobility. This data was available for separate locations, including grocery and pharmacy stores, recreation and retail stores, and workplaces. In addition, the cross-correlations between R_t_ and “#socialdistancing” mentions on Instagram and Twitter were also performed. The coefficient of determination (ρ^2^) was calculated and graphed, which represents the strength of the correlation at different time delays between R_t_ and each of the social mobility and social media measures. The peak of the coefficient of determination for each of these measures were tabulated along with the delay for which the greatest strength of relationship was found.

## Results

**Figure 1:**
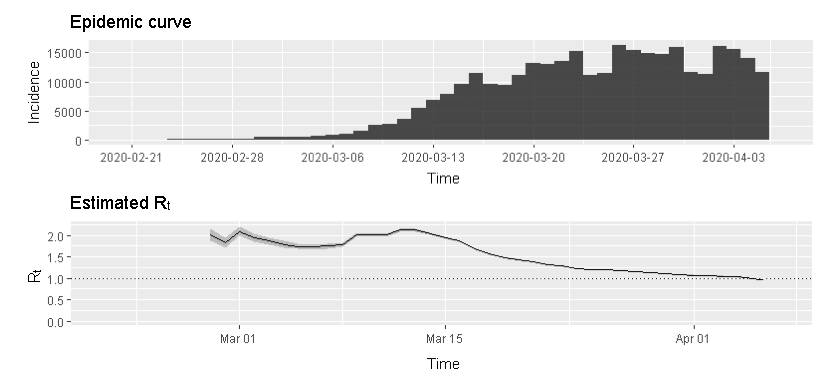
The time-variant or effective reproduction number (R_t_) represents the mean number of secondary cases generated by a primary case over a sliding weekly window. The estimated R_t_ is shown for the period of February 28 to April 5, 2020, calculated from the number of COVID-19 cases by symptom onset, with a mean serial interval of 3.96 days and SD of 4.75 days. The shaded error bands are equal to 1 SD of the estimated R_t_ for each date.

In Figure 1, the estimated R_t_ is shown for the period of February 28 to April 5, 2020, calculated from the number of COVID-19 cases by symptom onset, with a mean serial interval of 3.96 (SD 4.75) days. The shaded error bands are equal to 1 SD of the estimated R_t_ for each date.

**Figure 2:**
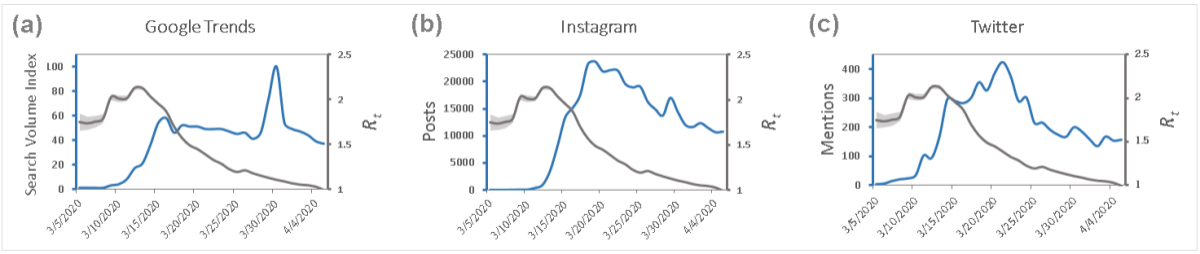
The left-most graph (a) shows the relationship between Google Trends search volume index (SVI) for “social distancing” and estimated R_t_ for the United States over the time period from March 5 to April 5, 2020. The light blue line represents the Google SVI and the dark grey line represents the estimated R_t_. The middle and right-most graphs show the relationships between estimated R_t_ and total social media mentions for “#socialdistancing” on (b) Instagram and (c) Twitter over the same time period. Light blue lines refer to total number of mentions for "#socialdistancing" and dark grey lines represent the estimated R_t_. Error bands shown in light gray shading represent 1 SD from the mean R_t_ calculated at each date. R_t_: time-varying reproduction number.

Significant negative correlations were found between the Google SVI for the search query “social distancing” and the R_t_ between the dates of March 5 and April 5, 2020, in the United States (*P*<.001). The relationship between estimated R_t_ and Google SVI is visualized graphically in Figure 2a. The strength of the correlation reached a peak at 4 days delay from the start of the searches when considering all cases in the United States, with a Pearson correlation coefficient of 0.72 (*P*<.001).

There was a total of 376,067 “#socialdistancing” mentions on Instagram and 6470 on Twitter in the studied time period. The increase in “#socialdistancing” mentions on Twitter and Instagram predate the appearance of a decrease in R_t_ seen in Figure 2b and c. The relationship between R_t_ and Instagram mentions (Figure 2c) is significant and strongest at a 4-day delay (*P*<.001) from the start of Instagram hashtag “#socialdistancing” mentions. Significance for Twitter is seen only at a 6-day delay (*P*<.001).

**Figure 3:**
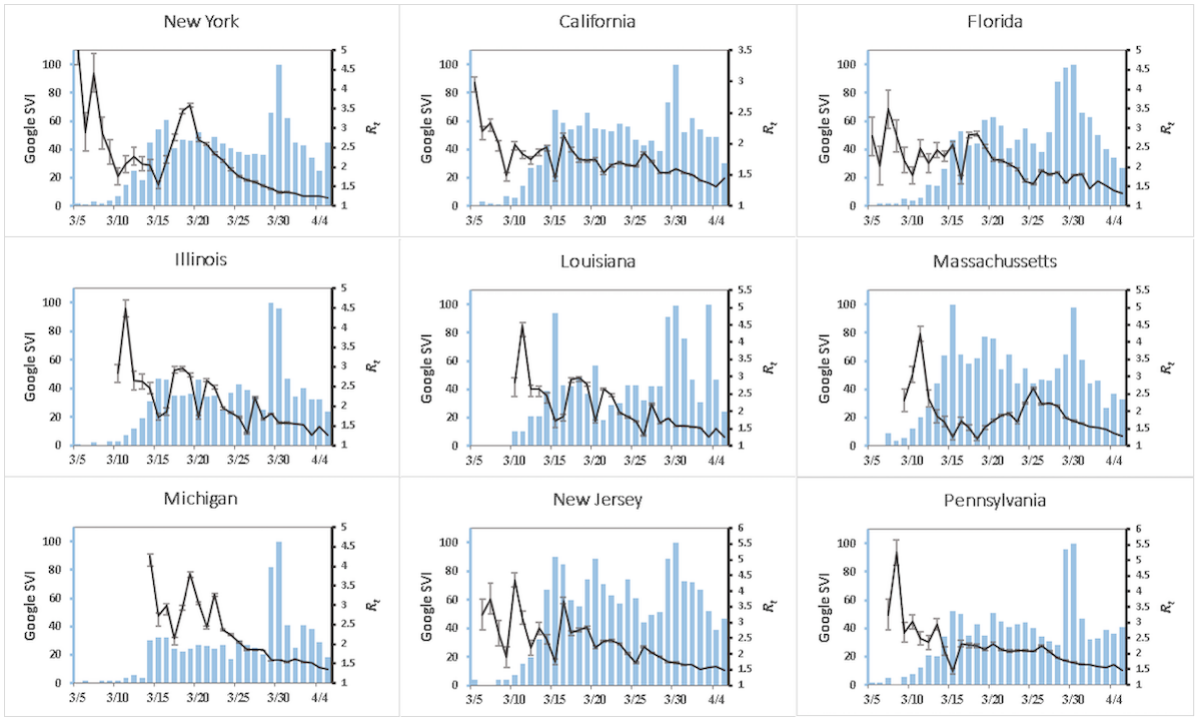
The estimated R_t_ was calculated individually for the top 9 states with the most confirmed COVID-19 cases on April 5, 2020. They are graphed for the period of March 5 to April 5 for each state (dark line) along with the Google search index for “social distancing” in each state for the same time period (light blue bars). Error bands represent 1 SD from the mean estimated R_t_ at each date. R_t_: time-varying reproduction number; SVI: search volume index.

When evaluated by state, New York, New Jersey, Massachusetts, Michigan, Pennsylvania, California, Louisiana, Illinois, and Florida all showed significant negative correlations between “social distancing” SVI and the state-specific R_t_ (*P*<.001; refer to Figure 3). These correlations reached peak significance at different delay periods. R_t_ for some states such as Massachusetts experienced an early correlation with increasing searches for “social distancing.” Other states such as New York and Louisiana experienced a larger time delay from the start of Google searches to a decrease in R_t_ at 6 and 8 days, respectively. Most states experienced a delay varying between 3-8 days before reaching peak significance.

**Figure 4:**
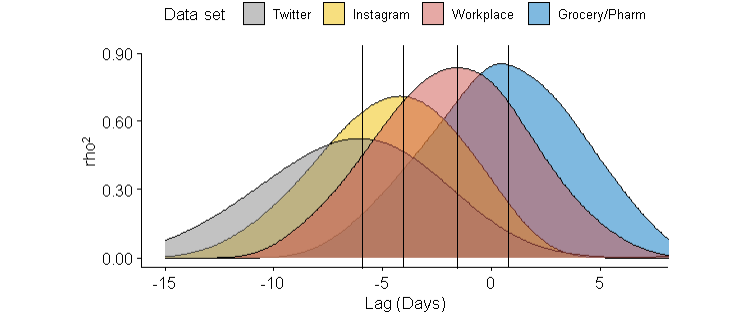
Cross-correlations (represented by ρ2, the coefficient of determination) between estimated R_t_ and social mobility changes, and social media mentions in the United States. The black lines represent the peak correlation. Social mobility changes include traffic at grocery, pharmacy, and workplace locations based on Google Maps tracking. Social media measures include mentions of “#socialdistancing” on Instagram and Twitter. Pharm: pharmacy; R_t_: time-varying reproduction number.

Significant correlations between R_t_ and social media appear to manifest themselves earlier in time when compared to social mobility measures, with peaks at –6 and –4 days for the relationship between R_t_ and Twitter and Instagram mentions, respectively (*P*<.001; refer to Figure 4). Social mobility correlated best with R_t_ at –2 days and +1 day for workplace and grocery/pharmacy, respectively.

The relationship between R_t_ and social media or social mobility (*P*<.001) reaches its strongest point at different delay periods, tabulated in Table 1. The increase in social media mentions predates the decrease in R_t_ the earliest, with a lag time of 4-6 days. Social mobility data also predate the decrease in R_t_, although at later times of 0-3 days. Table 1 also shows the strength of correlation between each of the measures and R_t_, represented by ρ^2^. The strongest correlations are between social mobility data and R_t_ with comparatively lower correlations between social media and R_t_. Google Trends, however, shows a comparable ρ^2^ with data from Apple Maps but not Google Maps, which exhibits the strongest correlations for all domains except parks.

**Table 1:** Peak correlations between social media and social mobility measures and associated time delay

Data set		ρ^2^	Lag (days)
**Social media**			
	Instagram	0.68	–4
	Twitter	0.47	–6
	Google Trends	0.72	–4
**Apple Maps**			
	Driving	0.75	–3
	Transit	0.80	–3
	Walking	0.73	–2
**Google Maps**			
	Grocery/pharmacy	0.89	+1
	Transit	0.83	–2
	Workplace	0.86	–2
	Parks	0.66	–2
	Recreation	0.84	0
	Residential	0.85	–2

## Discussion

### Principal Findings

In our study, we found that increased social distancing mentions on social media correlated with reduced US R_t_, with Google Trends correlating with reduced state-specific R_t_ as well. We also found that the correlation varied when social distancing mentions or search queries were lagged by a few days; this effect depended on the state and social media platform. The delay to reach peak strength discrepancy between Instagram and Twitter is interesting because the reach of Instagram in the United States is much greater, indicating possible time-sensitive influence on behavior imparted by user reach. Why the delay periods differed between states is unclear but may be partly explained by the unequal implementation of top-down public health interventions.

Instagram and Twitter mentions of “#socialdistancing” correlated earlier with reduced COVID-19 R_t_ in the United States than did social mobility measures from Google and Apple Maps. Interestingly, Twitter showed the earliest correlation with R_t_ but also has the lowest coefficient of determination. This finding may be explained by the fact that Twitter reaches the smallest user base compared to Instagram or Google Maps. Social media in general exhibited a weaker correlation with R_t_. This is expected since social mobility measures directly relate to the density of people congregating in an area, whereas social media is an indirect measure of social distancing and likely represents a smaller proportion of the population. Nonetheless, these findings confirm our hypothesis that social media may serve as earlier indicators of future social behavior.

The idea that lagging social distancing efforts as captured by social media produces significant reductions in R_t_ implicates a predictive role for social media. This is consistent with the interpretation that Google Trends, Instagram, and Twitter model the dissemination of information that may lead to individual decisions to undergo social distancing. Although the strength of the correlation for social media was found to be weaker than that for social mobility, the value was in the relationship of the correlation to time. Furthermore, the strength of the correlation may improve with subsequent studies using more accurate measures of social distancing in the media to actual social distancing behavior in the public.

An additional interpretation for the significance found in lagging social media mentions is that the delayed drop in R_t_ is also consistent with the expectation that social distancing is a method of primary prevention, as early practice prevents a future increase in R_t_. It is tempting to consider whether these effects depend on the incubation period for COVID-19. About 50% of infected individuals show symptoms by 5 days, and 97.5% by 12 days [2,31]. Our study shows that all 9 states exhibited a significantly reduced R_t_ with an 8-day lag period for social distancing search interest, supporting a quarantine time frame that confidently covers the upper limit of the incubation period. On the contrary, quarantine times closer to the median incubation period of COVID-19 may be insufficient, as only 30% of the states showed significant reductions in R_t_ when the lag period was shorter than the median incubation period. A parallel can be drawn from these findings, albeit speculatively: there may also be a threshold in this pandemic’s trajectory in the United States before which a termination of social distancing efforts may be too early.

### Limitations

Whether this proves valuable in the creation of more accurate assessments of the early epidemic course is uncertain due to limitations. Limitations of this study are inherent to the use of Google Trends, Instagram, and Twitter because they are presumably indirect measures of public behavior. The data represents a subtotal amount of mentions on Instagram and Twitter, and the study period is short and during the early course of the epidemic where testing and reporting COVID-19 was imperfect. Additionally, we focused on only the top nine states by incidence; although this was an effort to reduce false-positive findings from unreliable low-incidence states, it does introduce barriers to generalizing results to other states. Furthermore, social media may represent a biased sample of those that are internet literate and with access to internet, which may effectively covary with socioeconomic status, education, geography, and age.

### Conclusion

Our study demonstrates the utility of Google Trends, Instagram, and Twitter as epidemiological tools in the assessment of social distancing measures in the United States during the early course of the COVID-19 pandemic. Their correlation and earlier rise and peak in correlative strength with R_t_ when compared to social mobility may provide proactive insight into whether social distancing efforts are sufficiently enacted. Whether these findings translate to the hypothesized clinical value is uncertain due to limitations. Although social media remains a candidate to gauge the success of this containment measure in the early epidemic period, future studies should investigate how social media reactions change during the course of the epidemic and whether these correlation patterns with R_t_ persist.

## Abbreviations

R_t_: time-varying reproduction number
R_0_: basic reproductive number
SVI: search volume index
